# The complete genome of the tospovirus *Zucchini lethal chlorosis virus*

**DOI:** 10.1186/s12985-016-0577-4

**Published:** 2016-07-07

**Authors:** R. N. Lima, A. S. De Oliveira, M. O. Leastro, R. Blawid, T. Nagata, R. O. Resende, F. L. Melo

**Affiliations:** Laboratory of Virology, Department of Cell Biology, University of Brasilia, Brasilia, DF 70910-900 Brazil

**Keywords:** Zucchini, Tospovirus, Illumina, Genome, ZLCV

## Abstract

**Background:**

*Zucchini lethal chlorosis virus* (ZLCV) causes significant losses in the production of cucurbits in Brazil. This virus belongs to the genus *Tospovirus* (family *Bunyaviridae*) and seems to be exclusively transmitted by *Frankliniella zucchini* (Thysanoptera). Tospoviruses have a tripartite and single-stranded RNA genome classified as S (Small), M (Medium) and L (Large) RNA_S_. Although ZLCV was identified as a member of the genus Tospovirus in 1999, its complete genome had not been sequenced until now.

**Findings:**

We sequenced the full-length genome of two ZLCV isolates named ZLCV-SP and ZLCV-DF. The phylogenetic analysis showed that ZLCV-SP and ZLCV-DF clustered with the previously reported isolate ZLCV-BR09. Their proteins were closely related, except the non-structural protein (NSm), which was highly divergent (approximately 90 % identity). All viral proteins clustered similarly in our phylogenetic analysis, excluding that these ZLCV isolates have originated from reassortment events of different tospovirus species.

**Conclusion:**

Here we report for the first time the complete genome of two ZLCV isolates that were found in the field infecting zucchini and cucumber.

**Electronic supplementary material:**

The online version of this article (doi:10.1186/s12985-016-0577-4) contains supplementary material, which is available to authorized users.

## Body of text

*Zucchini lethal chlorosis virus* (ZLCV) is a member of the genus *Tospovirus*, family *Bunyaviridae* [1]. Although some tospovirus species are notorious for their broad-host range [2], ZLCV mainly infects cucurbits and is only known to be transmitted by *Frankliniella zucchini* (Thysanoptera: Thripidae) [3]. So far ZLCV has just been reported in Brazil, naturally infecting sponge gourd, West Indian gherkin, cucumber, watermelon and several species of squash [4, 5]. Infection by ZLCV abrogate fruit production in zucchini plants and the only resistant cultivar is *Cucurbita maxima* cv. Exposição [6], making ZLCV one of the most economically important viral pathogen for cucurbits in Brazil.

Tospoviruses have a tripartite single-stranded RNA genome and each segment is named according to its size. The L (large) RNA has a negative polarity and encodes a RNA-dependent RNA polymerase (RdRp) [7]. The ambisense M (medium) RNA encodes the precursor of two viral glycoproteins (Gn/Gc) and a non-structural protein (NSm) involved in viral cell-to-cell movement [8]. The S (small) RNA, which is also ambisense, encodes another non-structural protein (NS_S_) with RNA silencing suppression activity and the nucleocapsid (NP) protein [9].

There are currently 11 approved and 18 tentative tospovirus species, but only a small number of species were completely sequenced. Genome sequence data has the potential to solve key questions in tospovirus evolution, epidemiology and physiology, such as the occurrence and importance of interspecific reassortment [10] and the presence of potential undescribed genes [11]. Even though ZLCV was described in 1999 [1] and some genes of one isolate has been sequenced [12–14], its complete genome is still unknown. Here, we report the complete genome of two ZLCV isolates found infecting zucchini (*Cucurbita pepo* cv. Caserta) and cucumber (*Cucumis sativus* L.) in Brazil and compared them to other tospoviruses.

In 2010, a virus isolate (hereafter ZLCV-SP) from zucchini was found in a commercial field in São Paulo state and transmitted to *Datura stramonium* L. by *F. zucchini* as previously described [3]. Then, virus particles were propagated in *D. stramonium* by mechanical inoculation and infected leaves were used for ribonucleoprotein (RNP) purification following the protocol of De Avila et al. [15]. Moreover, cucumber plants showing typical ZLCV symptoms were collected in Planaltina, Federal District, in 2015. Viral particles were semi-purified from leaves as previously described [16]. Briefly, 40 g of leaf material were homogenized in PBS-EDTA plus 0.2 % 2-mercaptoethanol. The plant extract was then filtered and centrifuged through a sucrose cushion at 33,000 x g for 2 h and the pellet resuspended in PBS. Genomic RNA was extracted from purified RNP_S_ of both isolates as previously described by De Oliveira et al. [17] and sequenced at Macrogen (South Korea) using Illumina HiSeq 2000 platform. The resulting paired-end reads were filtered and assembled *de novo* using CLC Genomics Workbench version 6.0.3. The contigs related to ZLCV were selected using Blastx against a RefSeq virus database. To determine if the entire length of each segment was included in the assembled contigs, the reads were mapped back to the ZLCV related contigs. All contigs from both isolates presented the consensus sequences AGAGCAAU and AUUGCUCU at the 5’- and 3’-terminal ends, but some contigs presented distal terminal bases that were trimmed off. These palindromic sequences are conserved among all tospoviruses. Moreover, the ZLCV segments derived from cucumber samples (hereafter ZLCV-DF) presented some unresolved gaps (L segment: 1 gap of 13 nucleotide and 1 of 2 nucleotide). The genome of both isolates were annotated and submitted to NCBI GenBank under the accession numbers no. KU641378-KU641380 (ZLCV-SP) and KU681010-KU681012 (ZLCV-DF).

**Fig. 1:**
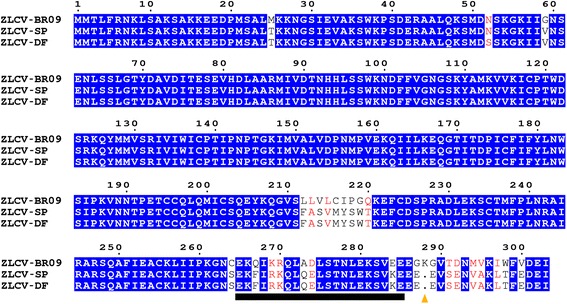
The NSm amino acid sequence alignment of ZLCV isolates. Residues common to the three sequences are shown in white on blue and similar residues are shown in red. The yellow triangle indicates the single amino acid deletion and the black rectangle indicates the coiled-coil (E^265^-E^285^) predicted structure (Uniprot Accession code: Q91PB4). This figure was made with ESPript (http://espript.ibcp.fr/ESPript/ESPript/)

Despite few variations, both genomes presented the same characteristics as listed in Table 1. The 5’ and 3’ UTR regions of both viruses presented the same size, except for one nucleotide in the 5’UTR of L segment of ZLCV-DF. The intergenic regions (IGR) presented small indels, for example, a duplication of 20 nucleotides in the M segment IGR of ZLCV-SP. Despite the importance of these regions as transcription termination signal [18], the impact of such deletions is still unclear. The L segments vary one nucleotide (as described above) between the isolates, 8,885 nt for ZLCV-SP and 8,886 nt for ZLCV-DF and encode a RdRp of about 331 kDa (2877 aa). The M segment contains 4,860 nt for ZLCV-SP and 4,829 nt for ZLCV-DF, and presented the ambisense arrangement typical of tospoviruses. For both isolates, an NSm protein of about 34 kDa (303 aa) and glycoprotein precursor (GP) protein of about 127 kDa (1136 aa) are predicted. The S segment is 3,524 nt long and encodes the NS_S_ and NP proteins with predicted molecular sizes of about 53 kDa (468 aa) and 29 kDa (261 aa), respectively. Since some genes of other ZLCV isolate (ZLCV-BR09) have been previously sequenced [12–14], their encoded proteins were compared with ZLCV-SP and ZLCV-DF (Additional file 1: Table S1). Pairwise sequence comparison of the NP amino acids indicates that ZLCV-SP is most closely related to the ZLCV-DF than ZLCV-BR09. The identities between the NP of ZLCV-SP/ZLCV-DF and between ZLCV-SP/ZLCV-BR09 are 99.23 % and 98.46 %, respectively, while the identity of ZLCV-DF/ZLCV-BR09 is 97.69 %. The NSm from ZLCV-BR09 displayed a significant variation in comparison with ZLCV-SP and ZLCV-DF (approximately 90 % identity) (Additional file 1: Table S1). Interestingly, most variation was located at the C-terminus with an increased number of non-conservative amino acid changes (Fig. 1), suggesting that this region may be under positive selection. Actually, it was recently demonstrated that this same region is implicated in NSm and plasma-membrane interaction [19]. However, the biological impact of these variations remains to be determined.

**Table 1:** Genome comparison of ZLCV isolates

	ZLCV-SP	ZLCV-DF
L RNA		
L RNA full length (nt)	8885	8886
5’UTR (nt)	222 (1–222)^a^	223 (1–223)
L gene ORF (nt)	8631 (223–8853)	8631 (224–8854)
L protein (aa)	2877 (330.85 kDa)	2877 (331.16 kDa)
3’UTR (nt)	32 (8854–8885)	32 (8855–8886)
M RNA		
M RNA full length (nt)	4860	4829
5’UTR (nt)	113 (1–113)	113 (1–113)
NSm gene ORF (nt)	909 (114–1022)	909 (114–1022)
NSm protein (aa)	303 (34.4 kDa)	303 (34,37 kDa)
IGR (nt)	344 (1023–1366)	313 (1–1335)
GPs gene ORF (nt)	3408 (1367–4774)	3408 (1336–4743)
GP protein (aa)	1136 (127.58 kDa)	1136 (127.73 kDa)
3’UTR (nt)	86 (4775–4860)	86 (4744–4829)
S RNA		
S RNA full length (nt)	3524	3524
5’UTR (nt)	87 (1–87)	87 (1–87)
NSs gene ORF (nt)	1404 (88–1491)	1404 (88–1491)
NSs protein (aa)	468 (53.06 kDa)	468 (53.01 kDa)
IGR (nt)	1009 (1492–2500)	1009 (1492–2500)
NP gene ORF (nt)	783 (2501–3283)	783 (2501–3283)
NP protein (aa)	261 (29.22 kDa)	261 (29.24 kDa)
3’UTR (nt)	241 (3284–3524)	241 (3284–3524)

nt = nucleotides

^a^ = position in the genome

aa = amino acids

kDa = kilodaltons

IGR = Intergenic region

GP = glycoproteins precursor

**Fig. 2:**
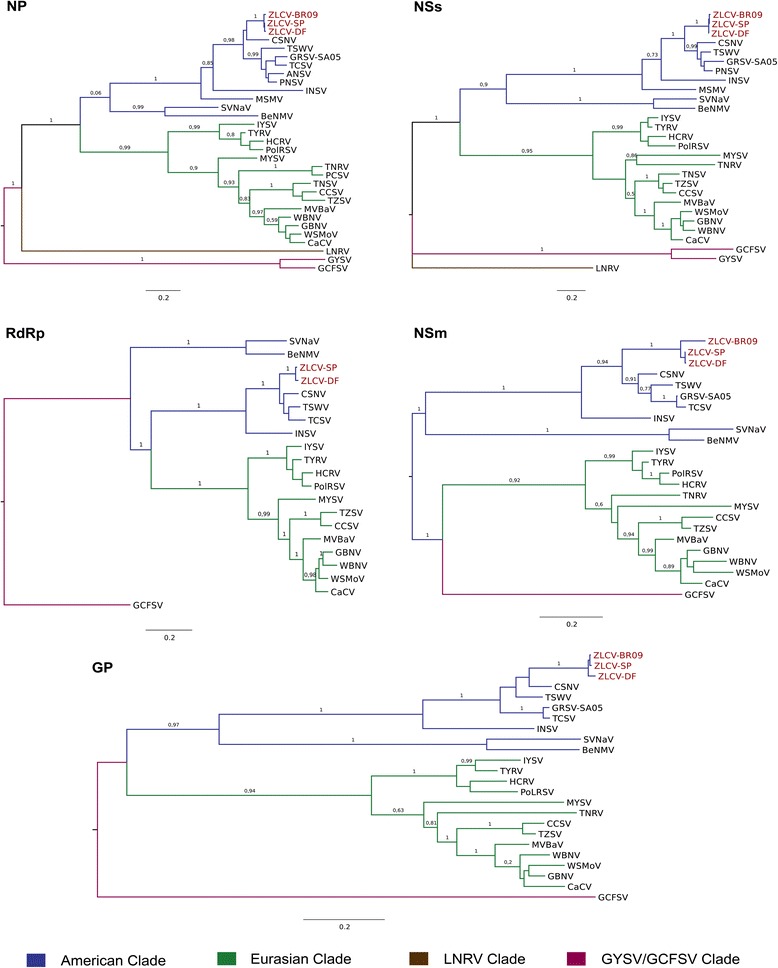
Maximum likelihood trees of tospoviruses inferred using all viral encoded proteins. All threes were midpoint rooted for clarity purposes. Fast tree support values are shown at the branches. The ZLCV isolates are labeled in red

To infer evolutionary relationships among tospoviruses, we compiled sequences of each tospovirus protein. The final data sets contain 33, 28, 23, 23 and 20 sequences for the NP, NSs, GP, NSm and RdRp proteins, respectively. The GenBank accession numbers are listed in Additional file 1: Table S2. The sequences were aligned using MAFFT [20] and the maximum likelihood trees were inferred using FastTree [21], both implemented in Geneious 9.1. As previously observed, tospoviruses can be divided into two geographic groups, with distinct viral species observed in Europe/Asia (Eurasian clade) and the Americas (American Clade) (Fig. 2). However, the analysis of NP and NSs trees indicate that some tospoviruses are clearly distinct from any species of these two groups, such as the Lisianthus necrotic ringspot virus (LNRV), Groundnut chlorotic fan-spot virus (GCFSV) and the *Groundnut yellow spot virus* (GYSV) (Fig. 2). The ZLCV isolates clustered within the American clade and no reassortment had occurred during the evolution of these ZLCV isolates (Fig. 2.).

Considering the high prevalence of tospoviruses worldwide, the complete genome of the ZLCV isolates is important for future surveillance and research. Additional investigations in important plant crops should keep being performed to extend the number of characterized species.

## Abbreviations

aa, amino acids; GP, glycoproteins precursor; IR, Intergenic region; kDa, kilodaltons; nt, nucleotides; RdRp, RNA-dependent RNA polymerase; RNP, ribonucleoprotein; ZLCV, *Zucchini lethal chlorosis virus*

## Additional file

Additional file 1: Table S1. Percent identity among ZLCV isolates proteins. **Table S2**. GenBank accession number of the sequences used in this study. (DOCX 61 kb)
