# Supplementary material for: The complete genome of the tospovirus Zucchini lethal chlorosis virus
Source: Virol J. 2016 Jul 7;13:123. doi: 10.1186/s12985-016-0577-4 (PMC4936248; doi:10.1186/s12985-016-0577-4)
Supplement: Additional file 1: Table S1. — Percent identity among ZLCV isolates proteins. Table S2. GenBank accession number of the sequences used in this study. (DOCX 61 kb) [file 12985_2016_577_MOESM1_ESM.docx]

Table S1. Percent identity among ZLCV isolates proteins

| NP | | | |
| --- | --- | --- | --- |
|  | ZLCV-BR09 | ZLCV-DF | ZLCV-SP |
| ZLCV-BR09 | 100% | 97.69% | 98.46% |
| ZLCV-DF | 97.69% | 100% | 99.23% |
| ZLCV-SP | 98.46% | 99.23% | 100% |
| NSs | | | |
|  | ZLCV-BR09 | ZLCV-DF | ZLCV-SP |
| ZLCV-BR09 | 100% | 98.72% | 99,14% |
| ZLCV-DF | 98.72% | 100% | 99.57% |
| ZLCV-SP | 99,14% | 99.57% | 100% |
| GP | | | |
|  | ZLCV-BR09 | ZLCV-DF | ZLCV-SP |
| ZLCV-BR09 | 100% | 97.53% | 99.30% |
| ZLCV-DF | 97.53% | 100% | 97.36% |
| ZLCV-SP | 99.30% | 97.36% | 100% |
| NS_M_ | | | |
|  | ZLCV-BR09 | ZLCV-DF | ZLCV-SP |
| ZLCV-BR09 | 100% | 90.73% | 91.03% |
| ZLCV-DF | 90.73% | 100% | 99.67% |
| ZLCV-SP | 91.03% | 99.67% | 100% |
| RdRp | | | |
|  | ZLCV-DF | ZLCV-SP |  |
| ZLCV-DF | 100% | 98.02% |  |
| ZLCV-SP | 98.02% | 100% |  |

Table S2. GenBank accession number of the sequences used in this study.

| **Acronym** | **Tospovirus** | **L RNA** | **M RNA** | **S RNA** |
| --- | --- | --- | --- | --- |
| ANSV | Alstroemeria necrotic streak virus | - | - | NP: GQ478668 |
| BeNMV | Bean necrotic mosaic virus | JF417980 | JN587269 | JN587268 |
| CaCV | Capsicum chlorosis virus | NC_008302 | NC_008303 | NC_008301 |
| CCSV | Calla lily chlorotic spot virus | FJ822962 | FJ822961 | AY867502 |
| CSNV | Chrysanthemum stem necrosis virus | KM114546 | KM114547 | KM114548 |
| GBNV | *Groundnut bud necrosis virus* | AF025538 | U42555 | AY871098 |
| GCFSV | Groundnut chlorotic fan- spot virus | KP146140 | KP146141 | AF080526 |
| GRSV-SA05 | *Groundnut ringspot virus* | - | NS_M_: AF213673 | NP: AF487516 |
|  |  |  | GP: AY574055 | NSs: JN571117 |
| GYSV | *Groundnut yellow spot virus* | - | - | HQ402596 |
| HCRV | Hippeastrum chlorotic ringspot virus | HG763861 | JX833565 | JX833564 |
| INSV | *Impatiens necrotic spot virus* | DQ425094 | DQ425095 | X66972 |
| IYSV | *Iris yellow spot virus* | FJ623474 | FJ361359 | AF001387 |
| LNRV | Lisianthus necrotic ringspot virus | - | - | AB852525 |
| MSMV | Melon severe mosaic virus | - | - | EU275149 |
| MVBaV | Mulberry vein banding associated virus | KM819698 | KM819699 | KM819701 |
| MYSV | Melon yellow spot virus | AB061774 | AB061773 | AB038343 |
| PCSV | Pepper chlorotic spot virus | - | - | KF383956 |
| PNSV | Pepper necrotic spot virus | - | - | HE584762 |
| PolRSV | *Polygonum ringspot virus* | KJ541746 | KJ541745 | KJ541744 |
| SVNaV | Soybean vein necrosis associated virus | HQ728385 | HQ728386 | HQ728387 |
| TCSV | *Tomato chlorotic spot virus* | HQ700667 | NS_M_:AF213674 | NP: AF282982 |
|  |  |  | GP: AY574054 |  |
| TNRV | Tomato necrotic ringspot virus | - | FJ947152 | FJ489600 |
| TNSV | Tomato necrotic spot virus | - | - | KM355773 |
| TSWV | *Tomato spotted wilt virus* | NC_002052 | NC_002050 | DQ915948 |
| TYRV | Tomato yellow ring virus | JN560178 | JN560177 | AY686718 |
| TZSV | Tomato zonate spot virus | EF552435 | EF552434 | EF552433 |
| WBNV | *Watermelon bud necrosis virus* | GU735408 | GU584185 | GU584184 |
| WSMoV | *Watermelon silver mottle virus* | AY863200 | DQ157768 | AB042650 |
| ZLCV-BR09 | *Zucchini lethal chlorosis virus* | - | NS_M_: AF213676 | NP: AF067069 |
|  |  |  | GP: AB274027 | NSs: JN572104 |
| ZLCV-DF | *Zucchini lethal chlorosis virus* | KU681010 | KU681012 | KU681011 |
| ZLCV-SP | *Zucchini lethal chlorosis virus* | KU641378 | KU641379 | KU641380 |
